# Evaluating the efficiency of primary health care institutions in China: an improved three-stage data envelopment analysis approach

**DOI:** 10.1186/s12913-023-09979-3

**Published:** 2023-09-15

**Authors:** Wanmin Su, Yatian Hou, Mengge Huang, Jiamian Xu, Qingfeng Du, Peixi Wang

**Affiliations:** 1 General Practice Center, The Seventh Affiliated Hospital, Southern Medical University, Foshan, 528244 Guangdong People’s Republic of China; 2 School of Nursing and Health, Henan University, Kaifeng, 475004 Henan People’s Republic of China; 3 School of Traditional Chinese Medicine, Southern Medical University, Guangzhou, 510515 Guangdong People’s Republic of China

**Keywords:** Primary health care institutions, Efficiency measurement, Super-efficiency SBM DEA model, Three-stage DEA model, Global benchmarking technique, External environment factors, China

## Abstract

**Background:**

Primary health care (PHC) institutions are key to realizing the main functions of the health care system. Since the new health care reform in 2009, the Chinese government has invested heavily in PHC institutions and launched favorable initiatives to improve the efficiency of such institutions. This study is designed to gauge the efficiency of PHC institutions by using 2012–2020 panel data covering 31 provinces in China.

**Methods:**

This study applied an improved three-stage data envelopment analysis (DEA) model to evaluate the efficiency of PHC institutions in China. Unlike the traditional three-stage DEA model, the input-oriented global super-efficiency slack-based measurement (SBM) DEA model is used to calculate the efficiency in the first and third stages of the improved three-stage DEA model, which not only allows the effects of environmental factors and random noise to be taken into account but also deal with the problem of slack, super-efficiency and the comparability of interperiod efficiency values throughout the efficiency measurement.

**Results:**

The results show that the efficiency of PHC institutions has been overestimated due to the impact of external environmental factors and random noise. From 2012 to 2020, the efficiency of PHC institutions displayed a downward trend. Moreover, there are significant differences in the efficiency of PHC institutions between regions, with the lowest efficiency being found in the northeast region. The efficiency of PHC institutions is significantly affected by residents’ annual average income, per capita GDP, population density, the percentage of the population aged 0–14, the percentage of the population aged 65 and older, the number of people with a college education and above per 100,000 residents, and the proportion of the urban population.

**Conclusions:**

Substantial investment in PHC institutions has not led to the expected efficiency gains. Therefore, more effective measures should be taken to improve the efficiency of PHC institutions in China based on local conditions. This study provides a new analytical approach to calculating the efficiency of PHC institutions, and this approach can be applied to efficiency evaluation either in other fields or in other countries.

**Supplementary Information:**

The online version contains supplementary material available at 10.1186/s12913-023-09979-3.

## Introduction

China's medical and health care development has made significant progress since the launch of the new health care reform in 2009. This progress includes achieving full medical insurance coverage, promoting the equalization of basic public health services, establishing an essential medicine system, and significantly advancing the health level and life expectancy of Chinese residents [1, 2]. According to the China Health Statistics Yearbook, the main health indicators are at the forefront of middle- and high-income countries, with life expectancy per capita increasing from 74.8 years in 2010 to 78.2 years in 2021, the maternal mortality rate decreasing from 30.0/100,000 to 16.1/100,000, the infant mortality rate decreasing from 13.1‰ to 5.0‰, and the under-five mortality rate declining from 16.4‰ to 7.1‰ [3, 4]. Despite these achievements, China's health care delivery system needs to avoid the risk of heading toward high cost and low value [2]. The Chinese health care service system is mainly composed of hospitals and primary health care (PHC) institutions, and they have a complementary relationship in terms of their designated functions. However, in reality, the medical and health services provided by hospitals and PHC institutions are poorly coordinated, with hospitals becoming one-stop providers of health services and the functional role of PHC institutions as gatekeepers becoming marginalized [5]. According to the China Health Statistics Yearbook, from 2012 to 2021, the proportion of PHC institution visits to total visits dropped yearly, with a decrease in the total volume of medical services from 59.7% to 50.2%. Additionally, the proportion of admissions to PHC institutions to total admissions fell from 23.6% to 14.5%. Conversely, the proportion of hospital visits rose from 36.9% to 45.8%, and the proportion of admissions to hospitals rose from 71.5% to 81.5% [3, 6]. When hospitals are always the first choice for residents to seek medical treatment, regardless of whether they are facing serious or minor illnesses, problems such as prolonged waiting times for patients, shortened doctor-patient interaction and communication, and increased medical costs will arise, which will deepen the conflicts between doctors and patients.

The Law of the People's Republic of China on Basic Medical Care and Health Promotion has clearly stipulated the basic position of the PHC system in the health care service system, and the government has invested heavily in PHC institutions to improve the efficiency of such institutions. For example, the Chinese government has strengthened the training of primary health personnel, and by the end of 2020, the central government had invested a total of 1.02 billion yuan to support the training of such personnel [7]. Moreover, since the 13th Five-Year Plan period (2016–2020), approximately 78 billion yuan in investment by the central government has been arranged to accelerate the standardization of PHC institutions and other projects [8]. In addition, in 2015, China started the construction of medical associations to promote the flow of high-quality medical resources from hospitals to PHC institutions, and by the end of 2019, more than 12,000 medical associations had been formed [9]. However, there is still a large discrepancy between the development of the PHC institutions and the health needs of people [10]. In 2022, the government once again clearly proposed continuing to boost investment in PHC institutions to improve the efficiency of PHC institutions and to strive to narrow the "gap" in the development of PHC institutions and hospitals [10]. The effect of a such a large amount of investment in PHC institutions is a problem that deserves much attention, and related research can serve as a reference for the international community. Therefore, this study uses the latest data to analyze the efficiency of PHC institutions.

Considering the efficiency measurement methods, data envelopment analysis (DEA) models are commonly used to measure the efficiency of PHC institutions due to the advantages of DEA. That is, DEA does not require a priori assumptions on the form of production functions, and DEA can be used to estimate the efficiency of decision-making units (DMUs) producing multiple outputs with multiple inputs [11–25]. However, when using DEA models, many studies have failed to consider the effects of the slack, environmental variables, random noise, the super-efficiency problem, and the comparability of results over time, leading to biased conclusions. This study aims to compensate for this deficiency and construct an improved three-stage DEA model that combines the advantages of the super-efficiency slack-based measurement (SBM) DEA model, the three-stage DEA model, and the global benchmarking technique (GBT) to evaluate the efficiency of PHC institutions in China at the provincial level from 2012 to 2020. In this way, the amount of slack, the super-efficiency problem, external environmental factors, statistical noise, and the comparability of results over time are taken into account during the efficiency evaluation process, making the results more precise. Our results provide a basis for developing policies tailored to local conditions to improve the efficiency of PHC institutions in different provinces in China. Additionally, this study provides a new analytical approach to calculating the efficiency of PHC institutions, and this approach can be applied to efficiency evaluation either in other fields or in other countries.

The remainder of this research is organized as follows. Section 2 conducts a literature review. Section 3 describes the methodology of the improved three-stage DEA model and presents the variables and data sources used in the improved model. Section 4 reports the results of the efficiency of PHC institutions at the provincial level in China, and it provides a comprehensive analysis of the empirical results. The major conclusions drawn from this research are presented in The second stage: tThe SFA model section.

### Literature review

Given that the low performance of PHC institutions can seriously affect the efficiency of the health care delivery system, in the past few years, scholars in China and elsewhere have conducted research on the efficiency of PHC institutions nationwide or in a particular region by applying DEA models. Such models are used in two major situations. One stream of the literature focuses on the efficiency evaluation of PHC institutions by using traditional DEA models and improved DEA models. Oikonomou et al. [13] applied a restricted DEA model to estimate the efficiency of rural Greek PHC institutions. Their results indicated that there was much room for efficiency improvement in the majority of rural PHC institutions. Cordero et al. [14] used extended DEA windows to assess 11 primary care units in the Basque Country from 2010 to 2013, and they demonstrated that the efficiency of these units improved during the study period. Liu et al. [15] used the super-efficiency SBM DEA model to analyze the efficiency of Chinese community health service institutions from 2008 to 2016 and revealed that community health service institutions in quite a few provincial areas were inefficient. Zhang et al. [16] quantified the efficiency and productivity of PHC institutions by using the traditional DEA model and the Malmquist productivity index (MPI) DEA model. Trakakis et al. [22] applied the MPI DEA model to study the productivity of 155 health centers in Greece, and the overall productivity factor was found to drop by 0.9% from 2016 to 2017 and by 5.2% from 2017 to 2018.

Another stream of the literature focuses on the efficiency evaluation of PHC institutions and on exploring the influencing factors through a two-stage DEA model, i.e., a combination of a DEA model and a regression model. Marschall and Flessa [11] assessed the efficiency of primary care in rural Burkina Faso by using the traditional DEA model, and they identified the reasons for inefficiency with the use of a truncated regression approach with a bootstrap procedure. Their research results showed that improvement in the accessibility of PHC institutions will have a significant impact on the performance of these institutions. Alhassan et al. [12] evaluated the efficiency of private and public PHC institutions in Ghana using the traditional DEA model in the first stage and then examined the factors associated with efficiency levels by employing a Tobit regression model in the second stage. Yitbarek et al. [17] adopted a two-stage DEA approach combining the use of traditional DEA models with the Tobit regression model to gauge the efficiency of neonatal health services in PHC institutions in Southwest Ethiopia and to identify the drivers of efficiency. They concluded that available resources could be utilized for additional newborns in the institutions, and a positive correlation was found between the years of experience of the health center head and the population served by the institutions and efficiency. Mohammadpour et al. [18] conducted an efficiency evaluation of rural PHC centers in Hamadan and explored the determinants of efficiency using the traditional DEA model and Tobit regression model. Zhong et al. [19] employed the traditional DEA model to estimate the efficiency of PHC institutions in Hunan Province, China, over the 2009–2017 period in the first stage and estimated the influencing factors using a Tobit regression model in the second stage. The results showed that the total population, the level of urbanization, and the proportion of beds exerted significant impacts on efficiency. Cao et al. [25] quantified the efficiency of basic public health services in Shandong Province, China, using the traditional DEA model and explored the influencing factors by means of a panel Tobit regression approach. Hou et al. [26] examined the efficiency of the hierarchical medical system, including PHC institutions and secondary and tertiary hospitals, by using the super-efficiency SBM DEA model. Then, the efficiency scores were regressed on a group of environmental and management factors through bootstrap truncated regressions.

The studies above can help us understand to some extent the performance of PHC institutions in China and other countries. However, based on the analysis of the available literature, there is still room for improvement in the methods used to assess the efficiency of PHC institutions. First, both traditional DEA models and two-stage DEA models fail to obtain efficiency values that exclude the effects of environmental factors and random noise. As a nonparametric estimator, DEA models are based on a finite sample of observations and do not consider the measurement errors in efficiency estimation, with the potential consequence of erroneous conclusions [27, 28]. Additionally, DEA models assume that DMUs are homogeneous in terms of the environment under which they operate [29, 30]. When DMUs are not homogeneous, the efficiency scores may reflect the underlying differences in environments rather than any inefficiencies. There are significant differences in the socioeconomic development of China's 31 provinces. Therefore, it is essential to control for the effects of environmental factors and random noise to obtain real efficiency values when measuring the efficiency of PHC institutions in China’s 31 provinces. The three-stage DEA method proposed by Fried et al. [31] can be applied to obtain efficiency values that exclude the effects of environmental factors and random noise, and it has been used for efficiency assessment in medical and health industry [21, 32, 33]. However, the model used to measure efficiency in the first and third stages of the three-stage DEA model is the traditional DEA model, which does not consider the slack and the super-efficiency problem [34–37]. Additionally, when using panel data to analyze dynamic changes in efficiency, another key issue should be fully considered, namely, the comparability of interperiod efficiency values. Contemporaneous benchmarking techniques or cross-benchmarking techniques are often used in the literature. However, as a result, efficiency scores measured in different periods lack interperiod comparability, and the reliability of the results can be affected [35, 36, 38, 39]. For slack and the super-efficiency problem, the super-efficiency SBM DEA model proposed by Tone [34] has been shown to be a good tool for addressing these two issues, and it has been applied for the efficiency measurement of Chinese community health service institutions [15], public hospitals [40], and hierarchical medical systems [26]. For the problem of the comparability of interperiod efficiency values, the GBT proposed by Pastor and Lovell [38] can address this issue by treating the same samples from different observation periods as different DMUs and combining them into one sample set, and it has been used extensively to evaluate efficiency [35, 36, 39]. To accurately measure the efficiency of PHC institutions, a better option is to consider the abovementioned issues in DEA models. However, thus far, this remains an unresolved problem. To fill this gap, this study constructs a global super-efficiency SBM DEA model that combines the advantages of the super-efficiency SBM DEA model and GBT to simultaneously address the issues represented by slack, the super-efficiency problem and the comparability of interperiod efficiency values. Then, the global super-efficiency SBM DEA model is used to measure the efficiency of PHC institutions in the first and third stages of the three-stage DEA model, different from the traditional three-stage DEA model, which uses the traditional DEA model to calculate efficiency in the first and third stages. This study refers to the improved model as an improved three-stage DEA approach. The improved three-stage DEA approach combines the advantages of the super-efficiency SBM DEA model, GBT, and three-stage DEA model, simultaneously taking into account slack, the super-efficiency problem, environmental factors, random noise, and the comparability of interperiod efficiency values in the efficiency measurement. This method can provide a feasible approach to measuring the real efficiency of PHC institutions or other health care institutions.

## Materials and methods

**Fig. 1:**
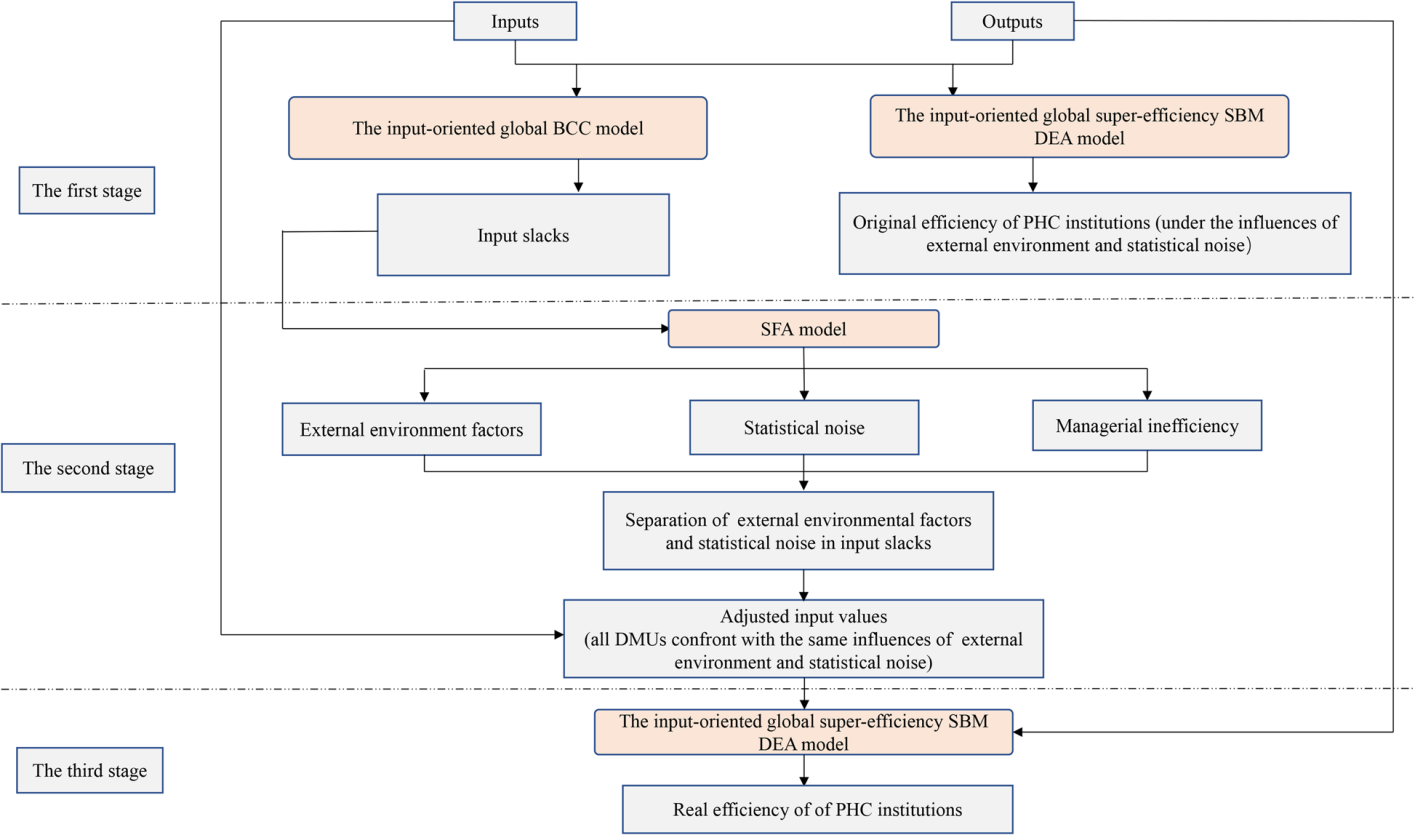
The methodological framework of the improved three-stage DEA model

The traditional DEA models include the CCR model proposed by Charnes, Cooper and Rhodes [41] based on the assumption of constant returns to scale and the BCC model proposed by Banker, Charnes and Cooper [42] based on the assumption of variable returns to scale. DEA models can be divided into two categories, i.e., input-oriented and output-oriented DEA models, in view of the relationship between inputs and outputs. Accounting for the nondiscretionary nature of outputs, i.e., the fact that health care facility managers have more control over inputs than outputs, such as the number of patients treated and the small elasticity of health care demand [16, 43], this paper utilizes the input-oriented global super-efficiency SBM DEA model to calculate efficiency. Additionally, because the data used are 2012–2020 panel data and the dependent variable of the stochastic frontier analysis (SFA) model in the second stage needs to be greater than or equal to 0, the input-oriented global BCC model is employed to calculate the slack of each input, similar to the approach taken by Fried et al. [31]. The methodological framework of this study is explained in Fig. 1. In the first stage, the original efficiency of PHC institutions under the influences of the external environment and stochastic disturbances are measured via the input-oriented global super-efficiency SBM DEA model. The input-oriented global super-efficiency SBM DEA model takes full advantage of the super-efficiency SBM DEA model and GBT, taking into account slack, the super-efficiency problem, and the comparability of interperiod results throughout the efficiency measurement. In the second stage, the SFA model is established by treating each input slack as a dependent variable and seven external environmental factors as independent variables, and the adjusted input values are obtained by excluding the effects of the external environment and random factors based on the SFA model. In the third stage, the real efficiency of PHC institutions, excluding the influences of the external environment and stochastic disturbances, is recalculated by means of the input-oriented global super-efficiency SBM DEA model using the adjusted input values and original outputs. The efficiency calculated in the first and third stages is technical efficiency (TE), which can be further decomposed into pure technical efficiency (PTE) and scale efficiency (SE) by using MaxDEA 8 Ultra software [44]. TE equals PTE multiplied by SE [45]. TE denotes the capacity to obtain the maximum possible output for a given input or to produce a given output with a minimum amount of input at the optimal production scale. PTE denotes the capacity to obtain the maximum possible output for a given input or to produce a given output with a minimum amount of input at the actual production scale, and reflects the levels of medical technology and management. SE denotes the difference between the actual production scale and the optimal production scale [44, 46].

### The first stage: the input-oriented global super-efficiency SBM DEA model and input-oriented global BCC model

Suppose that there are $n$ DMUs and that the observation period is $\tau =1,\cdots ,T$, with the input and output matrices being $X=\left({\times }_{kj}\right)\in {R}^{m\times n}$ and $Y=\left({Y}_{rj}\right)\in {R}^{s\times n}$ respectively. The input-oriented global super-efficiency SBM DEA model can be established as follows [34]:

$${\rho }_{it}^{{G}^{*}}=min\frac{1}{m}\sum_{k=1}^{m}{}^{{\overline{x} }_{kj\tau }}\!\left/ \!{}_{{x}_{kit}}\right.$$

subject to

1 $$\begin{array}{c}{\overline x}_{j\tau}\geq\sum\nolimits_{j=1(j\neq iif\tau=t)}^n\sum\nolimits_{\tau=1}^T\lambda_{j\tau}x_{j\tau}\\\begin{array}{c}{\overline y}_{j\tau}\leq\sum\limits_{j=1\left(j\neq iif\tau=t\right)}^n\sum\limits_{\tau=1}^T\lambda_{j\tau}y_{j\tau}\end{array}\\{\overline x}_{j\tau}\geq x_{it},{\overline y}_{j\tau}=y_{it},\lambda_{j\tau}\geq0\\\tau=1,2,\cdots,\mathrm T;j=1,2,\cdots,\mathrm n;k=1,2,\cdots,\mathrm m\end{array}$$

where ${x}_{kj\tau }$ and ${y}_{rj\tau }$ represent the $k$-th input variable and the $r$-th output variable of the $j$-th DMU in period $\tau$, respectively. ${\lambda }_{j\tau }$ is a nonnegative vector of the $j$-th DMU in period τ.

The input-oriented global BCC model can be established as follows [31, 47]:

$$min\theta$$

subject to

2 $$\begin{array}{c}\theta x_{kj\tau o}\geq\sum_{j=1}^n\sum_{\tau=1}^T\lambda_{j\tau}x_{kj\tau}\\\sum\limits_{j=1}^n\sum\limits_{\tau=1}^T\lambda_{j\tau}y_{rj\tau}\geq y_{rj\tau o}\\\sum\limits_{j=1}^n\sum\limits_{\tau=1}^T\lambda_{j\tau}=1\\\lambda_{j\tau}\geq0,\tau=1,2,\cdots,\mathrm T;j=1,2,\cdots,\mathrm n;\\k=1,2,\cdots,m;r=1,2,\cdots,s\end{array}$$

where the subscript of the evaluated producer’s data contains the letter “$o$”. The meanings of ${\lambda }_{j\tau }$, ${x}_{kj\tau }$, and ${y}_{rjt}$ are the same as those mentioned above.

### The second stage: The SFA model

The input slacks computed in the first stage demonstrate the disparities between the real and targeted inputs, impacted by three effects: managerial inefficiency, environmental influences, and statistical noise [31]. The SFA model, which considers the environmental variables as explanatory variables and the input slack variables as explained variables to control for the effects of nonmanagement factors on the input slacks, is established as follows:

3 $$\begin{array}{c}{S}_{ij}={f}^{i}\left({Z}_{j};{\beta }^{i}\right)+{v}_{ij}+{u}_{ij}, i=\mathrm{1,2},\cdots ,m; j=\mathrm{1,2},\cdots ,n\end{array}$$

where ${S}_{ij}$ is the slack value for the $i$-th input of the $j$-th DMU, derived from the first stage; ${Z}_{j}$ represents the vector of the environmental variables of the $j$-th DMU, ${Z}_{j}=({Z}_{1j}, {Z}_{2j}, \cdots , {Z}_{pj})$; $p$ is the number of environmental variables affecting the efficiency of PHC institutions; ${\beta }^{i}$ is the coefficient of every environmental variable; ${f}^{i}\left({Z}_{j};{\beta }^{i}\right)$ denotes the function of environmental parameters calculated by ${f}^{i}\left({Z}_{j};{\beta }^{i}\right)={Z}_{j}{\beta }^{i}$;${v}_{ij}+{u}_{ij}$ is the mixed error term; and ${v}_{ij}$ and ${u}_{ij}$ are the random noise component and managerial inefficiency term for the $i$-th input of the $j$-th DMU, respectively. Assuming that the distributions of ${v}_{ij}$ (${v}_{ij} \sim N(0,{\sigma }_{vi}^{2})$ and ${u}_{ij}$ (${u}_{ij} \sim {N}^{+}(0,{\sigma }_{ui}^{2})$) are independent from each other, ${\gamma }_{i}={\sigma }_{ui}^{2}/{(\sigma }_{ui}^{2}+{\sigma }_{vi}^{2})$ is defined. The paremeters (${\beta }^{i},{\sigma }_{i}^{2},{\gamma }_{i}$) are estimated using Frontier 4.1 software, and the inputs are adjusted in accordance with the regression results of the SFA model. The adjustment formula is as follows:

4 $$\begin{array}{c}{X}_{ij}^{A}={X}_{ij}+\left[max\left(f\left({Z}_{j};{\widehat{\beta }}_{i}\right)-f\left({Z}_{j};{\widehat{\beta }}_{i}\right)\right)\right]+\left[max\left({v}_{ij}\right)-{v}_{ij}\right], i=\mathrm{1,2},\cdots ,m; j=\mathrm{1,2},\cdots ,n\end{array}$$

where ${X}_{ij}^{A}$ is the adjusted input value and ${X}_{ij}$ is the initial input value in the first stage. The term $\left[max\left(f\left({Z}_{j};{\widehat{\beta }}_{i}\right)-f\left({Z}_{j};{\widehat{\beta }}_{i}\right)\right)\right]$ shows the adjustment assigning all DMUs under the most unfavorable environment confronted by DMUs. The component $\left[max\left({v}_{ij}\right)-{v}_{ij}\right]$ places all DMUs under a normal natural condition described as the most unfortunate environment confronted by DMUs. Such adjustments allow all DMUs to be under the same external environment and random noise.

### The third stage: the input-oriented global super-efficiency SBM DEA model

In this stage, the adjusted input values computed at the second stage and the original output data used at the first stage are utilized to recalculate the efficiency value of PHC institutions through the input-oriented global super-efficiency SBM DEA model. The efficiency scores obtained in the third stage are more accurate than those obtained in the first stage due to the elimination of the influence of external environmental factors and statistical noise.

### Variables and data sources

#### Input and output variables

The selection of appropriate inputs and outputs is essential to perform a meaningful analysis. On the basis of the literature on efficiency evaluation in the health care industry in China and elsewhere [1, 15, 21, 26, 44, 48–50], capital and labor are selected as the input indicators to measure the efficiency of PHC institutions. Specifically, beds represent capital investment, and doctors, nurses, and other health workers represent human resource investment. In addition, the output indicators should reflect the management objectives of the DMU [51]. To accelerate the construction of a hierarchical medical treatment pattern, increasing the volume of primary care treatment year by year has become one of the government's evaluation indicators for the performance of PHC institutions [10]. Based on the previous literature and policy objectives [1, 15, 19, 21, 26, 44, 48, 49, 52], the number of treatment visits and the number of admissions are treated as outputs. Therefore, the input-oriented global super-efficiency SBM DEA model used in the first and third stages of this research contains four inputs and two outputs, fulfilling the requirement that the number of DMUs must be greater than twice the total number of inputs and outputs (31 > 2 × (4 + 2)) to ensure the validity of the assessment results [51].

### Environmental variables

The efficiency of PHC institutions in Chinese provinces is influenced not only by internal inputs and outputs but also by external environmental factors, such as social and economic factors. Based on the studies of Yi et al. [53] and Auteri et al. [48], this paper selects seven indicators as external environmental variables in terms of the economy, population, and education. The seven indicators include residents’ annual average income, per capita GDP, the proportion of the urban population, population density, the percentage of the population aged 0–14, the percentage of the population aged 65 and older, and the number of people with a college education and above per 100,000 residents.

### Data sources

Based on the consistency and availability of indicators, the input and output data for PHC institutions in 31 provincial‒level regions in mainland China from 2012 to 2020 are selected as the sample for this study. All data used, including the data on the input‒output variables and external environmental variables, are collected from the “China Health Statistics Yearbook (2013–2021)” and the “China Statistical Yearbook (2013–2021)”. The descriptive statistics of the data on the input‒output variables and external environmental variables are presented in Table S1 and Table S2, respectively.

## Results and discussion

### The first stage: the original efficiency of PHC institutions

**Fig. 2:**
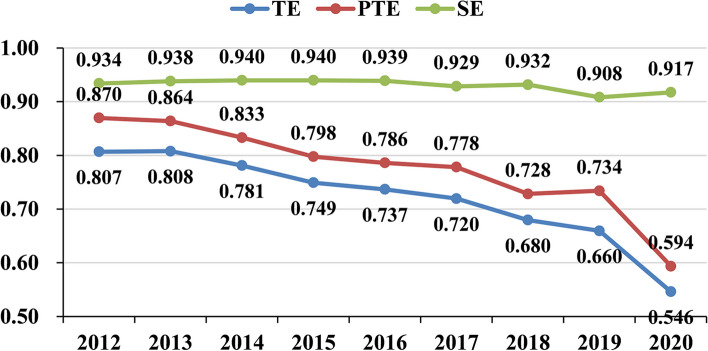
The trend in the efficiency of PHC institutions in stage 1 from 2012 to 2020

In this stage, the input-oriented global super-efficiency SBM DEA model in MaxDEA 8 Ultra software is used to calculate the efficiency scores of PHC institutions in 31 provinces in China from 2012 to 2020. The average scores of TE, PTE, and SE in the observation period were 0.721, 0.776, and 0.931, respectively (Table 1). SE was significantly greater than PTE, revealing that PTE has great potential for improvement, and this result is similar to that of Chen et al. [54], who calculated the efficiency of PHC institutions by using the traditional DEA model. It can be presumed that the inefficiency of PHC institutions in recent years was mainly due to the low levels of medical technology and management in PHC institutions. As a graphical supplement to the efficiency scores of PHC institutions in Table 1, Fig. 2 illustrates the dynamic changes in efficiency from 2012 to 2020. As shown, the TE of China’s PHC institutions decreased from 0.807 in 2012 to 0.546 in 2020, PTE decreased from 0.870 to 0.594, and SE decreased from 0.934 to 0.917, showing an unexpected downward trend. The downward trend in all three types of efficiency indicates that the growth in input in PHC institutions failed to generate efficiency gains. Although China has significantly expanded financial investment and introduced favorable policies to strengthen its PHC system, the development of PHC institutions still faces great challenges, such as inferior facilities and equipment, ineffective management, the poor capacity and skills of physicians at the primary level, and the inadequate construction level of information technology [5].

**Table 1:** The efficiency scores of PHC institutions in stage 1 from 2012 to 2020

Type	Region	2012	2013	2014	2015	2016	2017	2018	2019	2020	Mean
TE	Eastern region	0.870	0.889	0.895	0.874	0.858	0.817	0.776	0.761	0.616	0.817
	Central region	0.811	0.828	0.810	0.780	0.784	0.790	0.772	0.747	0.630	0.773
	Western region	0.823	0.801	0.738	0.695	0.674	0.662	0.617	0.601	0.514	0.681
	Northeast region	0.522	0.528	0.519	0.490	0.489	0.483	0.422	0.379	0.278	0.457
	Whole China	0.807	0.808	0.781	0.749	0.737	0.720	0.680	0.660	0.546	0.721
PTE	Eastern region	0.928	0.945	0.943	0.914	0.894	0.869	0.830	0.873	0.657	0.873
	Central region	0.833	0.853	0.834	0.807	0.809	0.820	0.793	0.771	0.644	0.796
	Western region	0.922	0.882	0.815	0.768	0.754	0.750	0.681	0.681	0.586	0.760
	Northeast region	0.541	0.546	0.538	0.510	0.509	0.506	0.449	0.408	0.310	0.480
	Whole China	0.870	0.864	0.833	0.798	0.786	0.778	0.728	0.734	0.594	0.776
SE	Eastern region	0.937	0.941	0.948	0.954	0.958	0.940	0.935	0.893	0.937	0.938
	Central region	0.974	0.972	0.972	0.968	0.971	0.965	0.972	0.967	0.974	0.971
	Western region	0.904	0.913	0.911	0.910	0.903	0.896	0.907	0.886	0.879	0.901
	Northeast region	0.965	0.965	0.964	0.959	0.958	0.952	0.939	0.928	0.895	0.947
	Whole China	0.934	0.938	0.940	0.940	0.939	0.929	0.932	0.908	0.917	0.931

### The second stage: analysis of the effects of external environmental factors and random noise

In the second stage, the SFA model is applied to separate the influences of the external environment, random noise, and managerial inefficiency on the input slack variables. The slack variables of inputs calculated in the first stage are treated as the dependent variables, and the seven external environmental variables (population density, the percentage of the population aged 0–14, the percentage of the population aged 65 and older, the number of people with a college education and above per 100,000 residents, the proportion of the urban population, residents’ annual average income, and per capita GDP) are taken as independent variables to construct the regression models. Table 2 displays the SFA estimation results, which are obtained using Frontier 4.1 software. First, the one-sided likelihood ratio (LR) value of each regression model obeys the mixed chi-squared distribution, and all results are significant at the 1% level, supporting the robustness of the SFA model. Second, the γ values of the four regression models are all close to 1 and significant at the 1% level, implying that low levels of medical technology and inefficient management were the main reason for the inefficiency of PHC institutions. Additionally, most regression coefficients of environmental variables on input slack variables are significant at the 1% level, and only a few are significant at the 5% or 10% level, which demonstrates that the environmental variables exert critical influences on the input slack variables. Thus, it is necessary to separate the influences of managerial factors, statistical noise, and environmental factors by using the SFA model to obtain the real efficiency of PHC institutions. In this model, the positive and negative signs of the regression coefficients denote different relationships between environmental indicators and input slack variables. Specifically, a positive regression coefficient implies that an increase in the value of an environmental variable will bring more input redundancy, with more resource waste and less efficiency. Conversely, a negative coefficient indicates that an increase in the value of an environmental variable will lead to less waste of inputs or more outputs. Next, the impacts of the environmental variables are analyzed separately.

**Table 2:** SFA estimation results in stage 2

Slack variables	Beds	Doctors	Nurses	Other health workers
Environmental variables	Coefficient value	Coefficient value	Coefficient value	Coefficient value
Constant term	-29,911.681^***^ (-16,079.181)	-9999.751^***^ (-4.438)	-9779.344^***^ (-6.526)	-6714.205^***^ (-427.018)
Population density	-2.213^*^ (-1.740)	-1.805^**^ (-2.188)	-1.351^***^ (-2.460)	-4.682^***^ (-3.090)
Percentage of the population aged 0–14	38,607.470^***^ (24,300.387)	-9115.881^***^ (-19.422)	16,103.214^***^ (84.075)	-36,155.125^***^ (-12,491.727)
Percentage of the population aged 65 and older	220,595.580^***^ (120,082.470)	122,461.640^***^ (257.617)	80,548.465^***^ (588.882)	125,587.110^***^ (13,043.921)
Number of people with a college education and above per 100,000 residents	0.202 (1.465)	0.235^***^ (2.461)	0.171^***^ (2.625)	0.211 (1.493)
Proportion of the urban population	191.922^***^ (4.448)	109.229^*^ (1.653)	51.008 (1.219)	111.931^***^ (2.614)
Residents’ annual average income	-0.364^***^ (-3.060)	-0.244^***^ (-2.941)	-0.156^***^ (-2.483)	-0.225^*^ (-1.821)
Per capita GDP	-0.032 (-0.707)	-0.055^*^ (-1.836)	-0.010 (-0.393)	-0.042 (-0.946)
${\sigma }_{i}^{2}$	111,446,850.000^**^(111,446,690.000)	85,915,979.000^***^ (66,028,501.000)	39,639,878.000^***^ (28,335,268.000)	158,777,230.000^***^ (158,775,080.000)
${\gamma }_{i}$	0.747^***^ (32.782)	0.831^***^ (56.641)	0.785^***^ (39.777)	0.856^***^ (65.981)
LR test	126.797^***^	200.480^***^	163.456^***^	232.217^***^

The data in brackets denote* t*-statistics *, ** and *** represent significance at the 10%, 5% and 1% levels, respectively

#### Population density

Population density has a significant (1%, 5%, 10% significance level) negative effect on the slack in beds, doctors, nurses and other health workers. This result reveals that PHC institutions located in areas with a higher population density are more inclined to reduce the redundancy of beds, doctors, nurses, and other health workers, thereby contributing to the efficiency of PHC institutions, which is consistent with the research results of Alatawi et al. [55], Tao et al. [56] and Zhang et al. [57]. In addition, the results of a study on the efficiency of health care expenditures showed that population density is positively associated with the efficiency of health care expenditures at the 1% significance level [58]. One possible reason is that the higher the population density is, the more concentrated the area in which the population resides, and the more convenient the supply of health care services, which reduces the cost of supply and increases the utilization of health care services, thus promoting the efficiency of PHC institutions.

#### Percentage of the population aged 0–14

The results show that the regression coefficients of the impact of the proportion of the population aged 0–14 are positive on the slack in beds and nurses and negative on the slack in doctors and other health workers. The coefficients are all statistically significant at the 1% level, suggesting that an increase in the proportion of the population aged 0–14 is highly correlated with the amount of input redundancy. A larger percentage of the population aged 0–14 will result in an increase in the slack in beds and nurses but a decrease in the slack in doctors and other health workers. Overall, its ultimate impact on the efficiency of PHC institutions is uncertain. Therefore, the impact of the percentage of the population aged 0–14 on the efficiency of PHC institutions needs to be confirmed by more studies.

#### Percentage of the population aged 65 and older

The coefficients of the impact of the proportion of the population aged 65 years and older on the amount of slack in the four inputs are positive and statistically significant at the 1% level. These results demonstrate that an increase in the proportion of the population aged 65 years and older will result in more redundancies of beds, doctors, nurses, and other health workers, thereby reducing the efficiency of PHC institutions. This finding is similar to that of Alatawi et al. [55] and Chen et al. [59]. The main impact of the aging population on the health care sector is the rapid increase in demand for health care. According to the sixth National Health Service Survey, in 2018, the two-week prevalence rate of people over 65 years old in China was 58.4%, an increase of 25.05% compared with 2013, and the hospitalization rate was 27.2%, the highest among all age groups [60]. The aging population has increased the demand for health services and the consumption of health resources in China, posing a great challenge to the country's health care service system. Therefore, in the context of an aging population, more efforts are needed to rationalize the allocation of health care resources and to improve management to promote the high-quality development of PHC institutions.

#### Number of people with a college education and above per 100,000 residents

Regarding the number of people with a college education and above per 100,000 residents, this indicator is positively correlated with the number of doctors and nurses at the 1% significance level. This result shows that the increasing number of people with a college education and above per 100,000 residents will lead to an increase in input redundancy or a decrease in output, thereby lowering the efficiency of PHC institutions. The main reason for this is that people with a college education and above are more likely to choose nonprimary care institutions when seeking medical care, as they have higher expectations for quality of care and the treatment environment [53, 61, 62]. Therefore, it is necessary to improve the medical technology and the treatment environment of PHC institutions to reinforce residents' confidence in such institutions and to attract more people to prefer these institutions when consulting medical services, thus enhancing the efficiency of PHC institutions and the efficiency of the entire health care delivery system.

#### Proportion of the urban population

The proportion of the urban population has a positive effect on the slack in all four inputs and is significantly (1%, 10% significance level) associated with the slack in beds, doctors, and other health workers. This result means that higher levels of urbanization will increase the redundancy of inputs and are thus not conducive to the efficiency of PHC institutions. This result is similar to that of previous studies [19, 53, 58, 63]. In general, the higher the level of urbanization is, the more health resources are invested [63, 64]. Although urbanization makes a significant contribution to the health of Chinese residents, as evidenced by a 0.37% increase in per capita life expectancy and a 2.48% decrease in the neonatal mortality rate for every 10% increase in the urbanization rate [64], it is still necessary to rationalize the investment of health resources.

#### Residents’ annual average income

Residents’ annual average income reflects the ability to pay for health care services to a certain extent. According to the results, the index is significantly (1%, 10% significance level) and negatively linked to the slack in beds, doctors, nurses, and other health workers. This result suggests that an increment in the average annual income of residents will diminish the redundant value of the input variables or increase the output, thus benefiting the efficiency of PHC institutions. A study on the factors influencing hospital efficiency in China also demonstrated a positive relationship between residents’ annual average income and hospital efficiency at the 1% significance level [65]. One possible explanation is that as the average annual income of residents increases, the potential demand for health care is released, and residents' health care expenditure rises correspondingly, thus contributing to a higher utilization of health care services.

#### Per capita GDP

Per capita GDP

Per capita GDP represents the economic basis of a region. Theoretically, the level of economic development is the main factor limiting the availability of medical and health resources in a region [66]. According to the regression results, the index has a negative impact on the four types of input slack and is significantly associated with the slack in doctors at the 10% level. An increase in per capita GDP will reduce the redundancy of inputs, thus contributing to the efficiency of PHC institutions. This result is consistent with that of previous studies [53, 58, 67, 68]. As a result of multiple factors, residents in economically developed areas may have higher health care needs, and therefore, more health resources are invested in developed areas [69, 70]. However, as people continue to migrate to developed regions, the total population in these regions continues to grow, and the demand for health services increases rapidly, resulting in a relative shortage in the supply of health resources. In this way, per capita GDP enhances the efficiency of PHC institutions in part by promoting the utilization of health services.

### The third stage: the real efficiency of PHC institutions

**Fig. 3:**
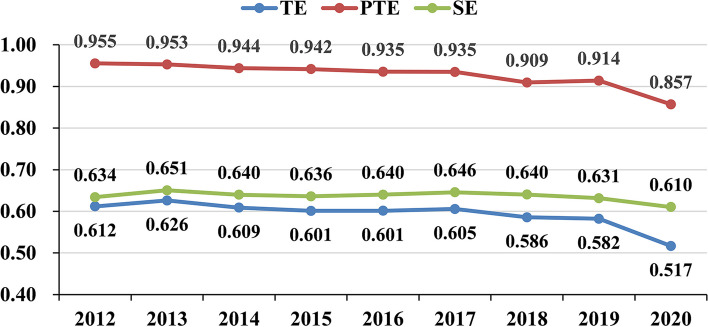
The trend in the efficiency of PHC institutions in stage 3 from 2012 to 2020

The real efficiency scores of PHC institutions in China from 2012 to 2020 computed based on the adjusted input data derived from the second stage are presented in Table 3. The average scores of TE, PTE, and SE are 0.593, 0.927, and 0.636, respectively. Here, PTE is significantly larger than SE, indicating that the inefficiency of PHC institutions in recent years was mainly due to the low level of SE. This result differs from the results of the first stage and of Chen et al. [54], who calculated the efficiency of PHC institutions by using the traditional DEA model. This difference indicates that not taking into account the effects of environmental factors and random noise can introduce bias into the results. From 2012 to 2019, all three types of efficiency showed a small decline, while a greater drop occurred between 2019 and 2020 (Fig. 3). The main reason for the greater decrease from 2019 to 2020 was the COVID-19 pandemic. To reduce the risk of cross-infection during the COVID-19 pandemic, medical and health institutions had strict requirements for patient access procedures, such as taking body temperature, showing travel codes and health codes, and providing 24-h or 48-h nucleic acid-negative certificates. In addition, the rigorous control measures of the pandemic caused people to be fearful of the new coronavirus and to develop the mindset of not going to a medical institution unless their condition was serious and urgent. These measures greatly reduced the outputs in 2020, including visits and admissions to PHC institutions.

**Table 3:** The real efficiency scores of PHC institutions in stage 3 from 2012 to 2020

Type	Region	2012	2013	2014	2015	2016	2017	2018	2019	2020	Mean
TE	Eastern region	0.647	0.658	0.661	0.666	0.667	0.662	0.640	0.643	0.555	0.644
	Central region	0.796	0.803	0.796	0.788	0.787	0.797	0.778	0.765	0.701	0.779
	Western region	0.538	0.559	0.517	0.498	0.496	0.504	0.491	0.492	0.447	0.505
	Northeast region	0.421	0.433	0.431	0.424	0.432	0.441	0.397	0.372	0.296	0.405
	Whole China	0.612	0.626	0.609	0.601	0.601	0.605	0.586	0.582	0.517	0.593
PTE	Eastern region	0.962	0.961	0.953	0.955	0.954	0.948	0.909	0.940	0.831	0.935
	Central region	0.941	0.948	0.940	0.937	0.938	0.943	0.928	0.916	0.862	0.928
	Western region	0.981	0.968	0.954	0.947	0.929	0.929	0.912	0.904	0.880	0.934
	Northeast region	0.858	0.875	0.883	0.887	0.895	0.899	0.866	0.863	0.841	0.874
	Whole China	0.955	0.953	0.944	0.942	0.935	0.935	0.909	0.914	0.857	0.927
SE	Eastern region	0.668	0.680	0.687	0.696	0.700	0.699	0.704	0.681	0.686	0.689
	Central region	0.833	0.836	0.835	0.829	0.827	0.833	0.825	0.818	0.800	0.826
	Western region	0.543	0.572	0.541	0.530	0.537	0.547	0.541	0.547	0.517	0.542
	Northeast region	0.490	0.494	0.487	0.476	0.481	0.489	0.457	0.430	0.353	0.462
	Whole China	0.634	0.651	0.640	0.636	0.640	0.646	0.640	0.631	0.610	0.636

In both the first and third stages, from 2012 to 2020, the efficiency of PHC institutions displayed a downward trend from, indicating that the relevant policies and measures used to strengthen the grassroots in recent years did not meet expectations. This result is similar to that of Xu et al. [71], meaning that there are still many unresolved issues in promoting the development of PHC institutions. For example, in the construction of medical associations, there are serious conflicts of interest between hospitals and PHC institutions, and hospitals and PHC institutions have not become a real community of responsibility, management, services and interests [72]. Thus, high-quality medical and health resources are not really sunk into PHC institutions. The problems of serious staff turnover in PHC institutions, the lack of quality health care personnel and relatively simple and outdated medical equipment are still prominent [73]. These problems have significantly limited the improvement in efficiency of PHC institutions in China. Therefore, in future reforms, relevant systems should be continuously established and improved to strengthen the deeper coordination between PHC institutions and hospitals, especially the alignment of economic interests [5]. Additionally, a reasonable performance incentive system, a title promotion channel, and social security measures should be explored to attract high-quality health professionals to work at the grassroots level, with a certain degree of autonomy granted to PHC institutions to give full play to their talent.

Compared with the first stage, the average value of the TE of PHC institutions decreased from 0.721 to 0.593, PTE increased from 0.776 to 0.927, and SE declined from 0.931 to 0.636 (Tables 1 and 3). PTE increased, while SE decreased significantly, resulting in a decrease in TE. This result means that external environmental factors have a marked impact on the efficiency of PHC institutions, especially on SE.

### Spatial distribution of the efficiency of PHC institutions

**Fig. 4:**
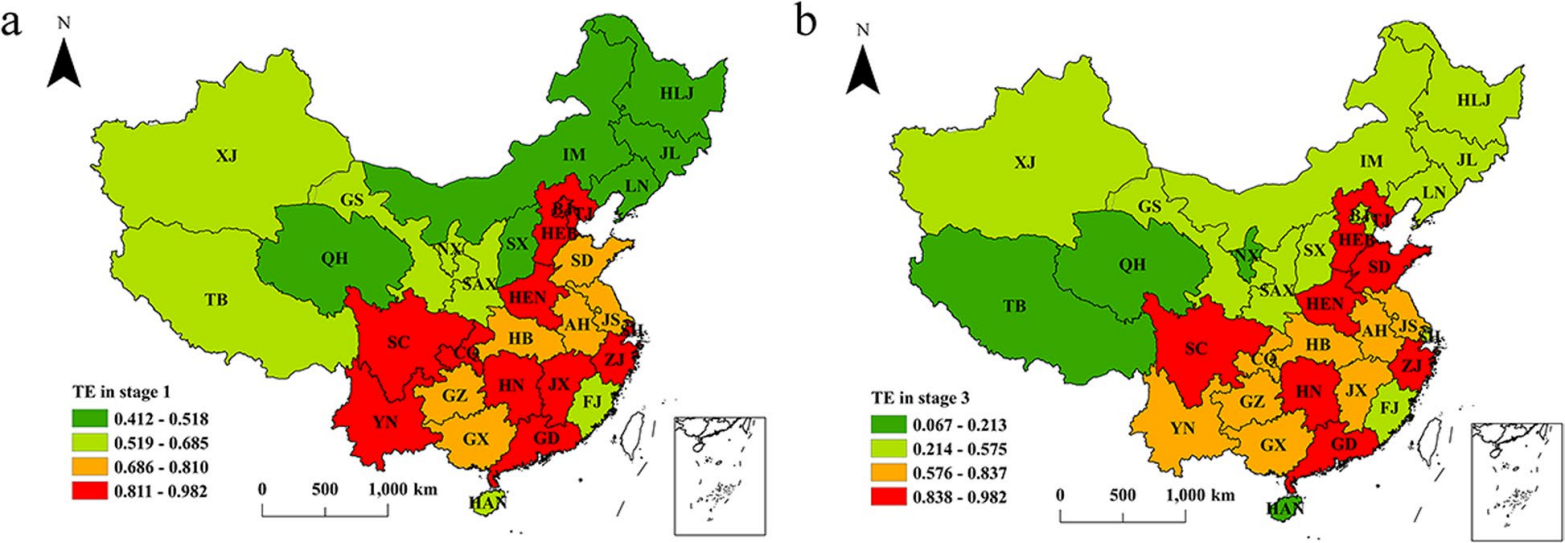
The average TE values of PHC institutions in China from 2012 to 2020

The natural breaks method [74] is used to divide the efficiency scores of PHC institutions in the first stage and the third stage from 2012 to 2020 into four grades. As shown in Fig. 4, from the first stage to the third stage, i.e., before and after the removal of environmental values and stochastic disturbances, the TE of PHC institutions in each region had certain changes, but the relative levels of TE in all regions did not differ much. The TE of PHC institutions also exhibited a certain clustering, as did the distribution of the TE of public hospitals [40], indicating that the efficiency of medical and health institutions in an area might be related to the efficiency of medical and health institutions in adjacent areas. The reasons for the clustering phenomenon may be related to the construction of regional medical centers and the competition among neighboring medical institutions. As the construction of regional medical centers progresses, medical and health institutions with high TE will lead and radiate to the medical institutions in surrounding areas, and the agglomeration phenomenon will become more obvious.

### Comparison of the regional efficiency of PHC institutions

**Fig. 5:**
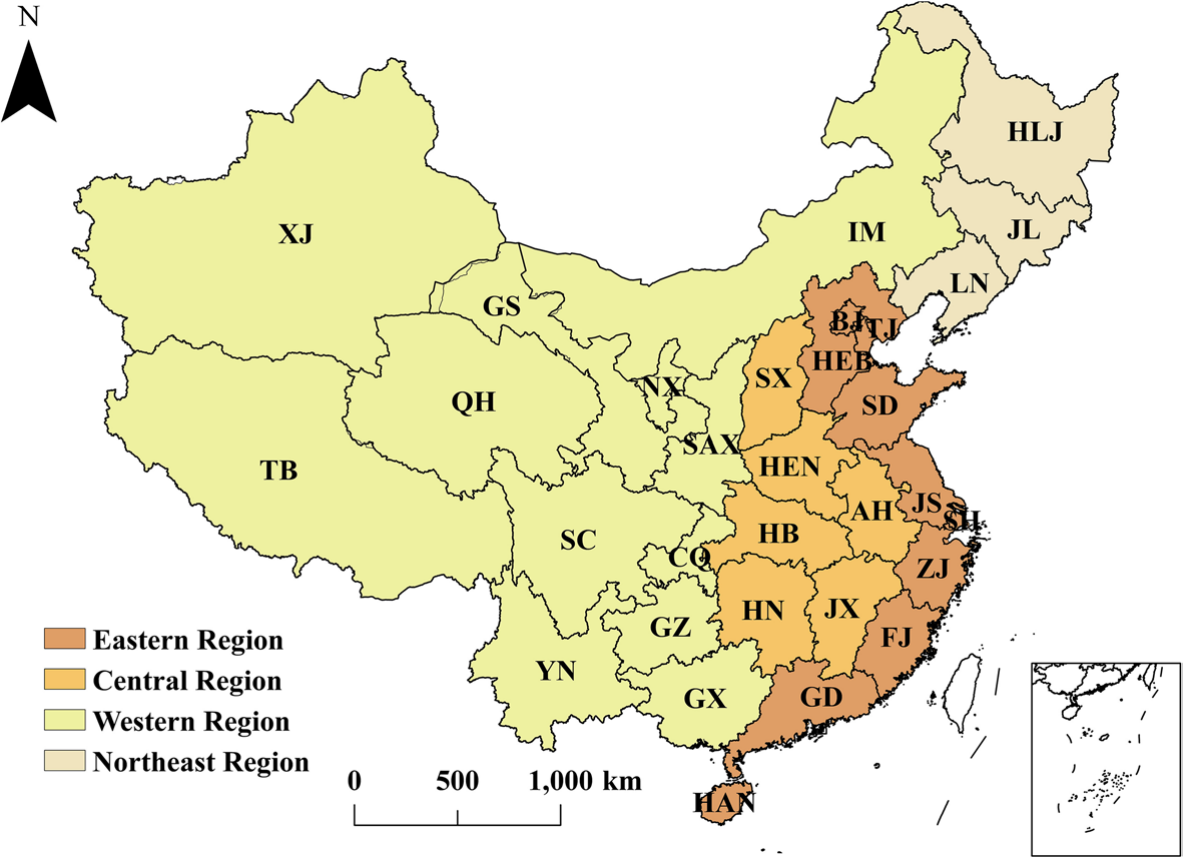
Four groups of regions in China

**Fig. 6:**
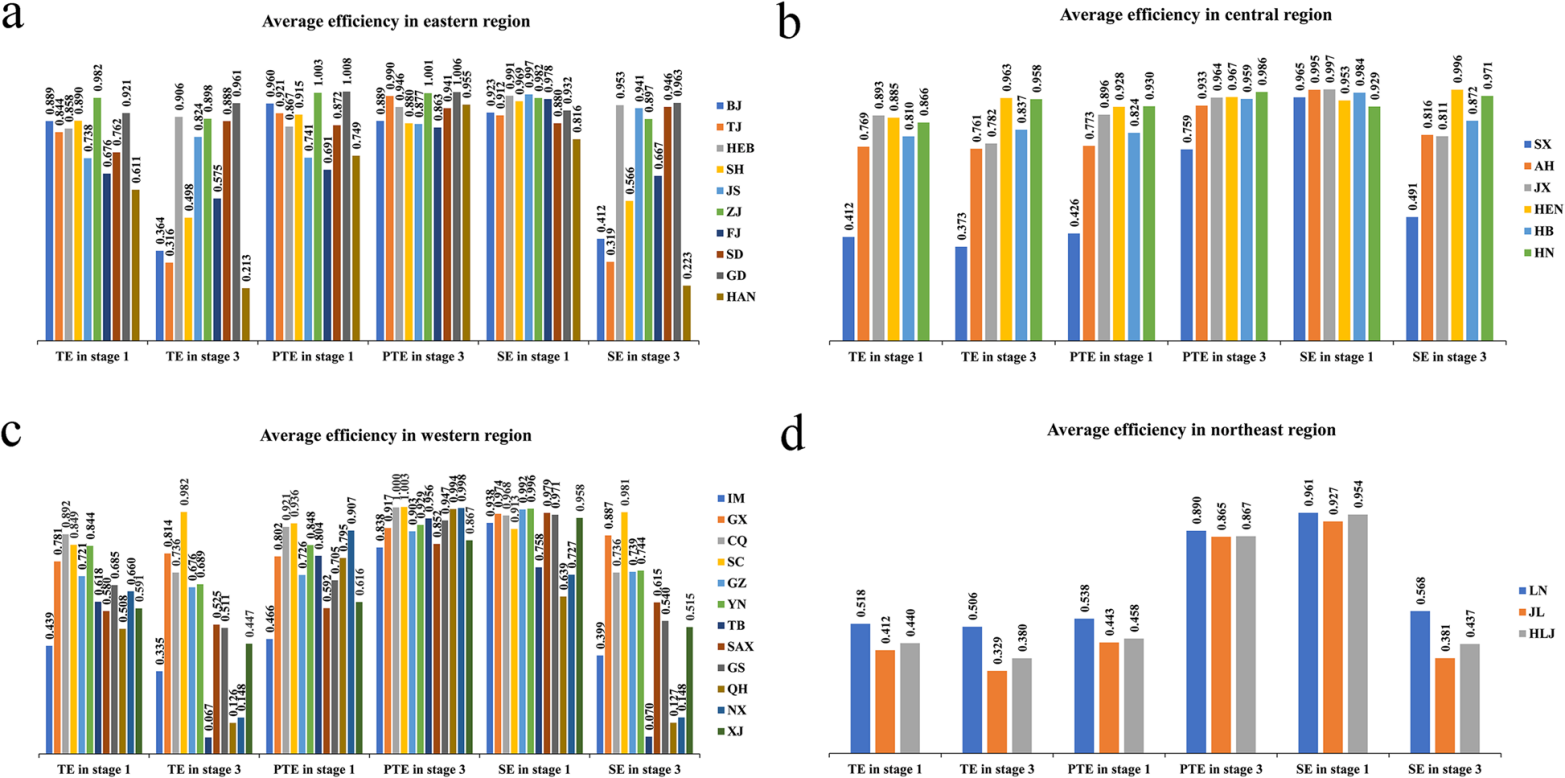
Three types of efficiency of PHC institutions in the four regions

To analyze the differences in the efficiency of PHC institutions across China, China’s 31 provinces are divided into four groups of regions based on their geographical location and economic development [75], as shown in Fig. 5. These groups are the eastern region (including Beijing (BJ), Tianjin (TJ), Hebei (HEB), Shanghai (SH), Jiangsu (JS), Zhejiang (ZJ), Fujian (FJ), Shandong (SD), Guangdong (GD), and Hainan (HAN)), the central region (including Shanxi (SX), Anhui (AH), Jiangxi (JX), Henan (HEN), Hubei (HB), and Hunan (HN)), the western region (including Chongqing (CQ), Shaanxi (SAX), Inner Mongolia (IM), Guangxi (GX), Sichuan (SC), Guizhou (GZ), Yunnan (YN), Tibet (TB), Gansu (GS), Qinghai (QH), Ningxia (NX), and Xinjiang (XJ)), and the northeast region (including Liaoning (LN), Jilin (JL), and Heilongjiang (HLJ)). Figure 6 illustrates the three types of efficiency for each region over the 2012–2020 period. As illustrated, changes in efficiency from the first stage to the third stage are evident, with a decrease in TE in most provinces (22 out of 31), indicating that the TE in these provinces is overestimated under the influence of environmental factors and random noise. Chen et al. [21] obtained different findings when measuring the TE of public hospitals using the traditional three-stage DEA model, concluding that most provinces (28 out of 31) had higher TE in the third stage. The application of different research methods and differences in the effects of the same environmental variables on the TE of public hospitals and PHC institutions may lead to different conclusions. Hence, more environmental variables should be explored in future studies to determine their impact on the efficiency of different health care institutions. Next, a comparative analysis of the efficiency of PHC institutions across regions is presented.

First, the TE of PHC institutions in GD in the third stage is 0.961, higher than that in other provinces in the eastern region. The highest TE of PHC institutions in GD is mainly due to its high level of economic development and innovation in management. As the most economically developed region in China, GD has ranked first in GDP for 34 consecutive years, providing a good material basis for the development of PHC institutions. Second, GD has actively created a new model for the management of PHC institutions and reformed the performance pay system of PHC institutions, which has greatly mobilized the enthusiasm of primary medical staff [76]. At the same time, to encourage high-quality health talents to serve in PHC institutions, GD has set up special positions for primary general practitioners, and it has offered generous welfare benefits. These measures have led to the high efficiency of PHC institutions in GD. Although PHC institutions in TJ have a PTE of 0.990 in the third stage, which is ahead of that of some other provinces, its SE is only 0.319, putting it well behind some other provinces in terms of TE. Compared with the first-stage results, the TE of PHC institutions in BJ, TJ, SH, HAN, ZJ and FJ shows a certain decline, with BJ and TJ experiencing larger declines of 0.525 and 0.528, respectively. These results indicate that the efficiency of PHC institutions in BJ and TJ is more influenced by environmental factors.

In the central region, the TE value of PHC institutions in SX in the third stage is 0.373, lagging far behind the values of the other five provinces. Chen et al. reached a similar conclusion when evaluating the TE of regional public hospitals and concluded that public hospitals in SX have the lowest TE [21]. Overall, the development of public hospitals and PHC institutions in SX is relatively backward, and the efficiency of public hospitals and PHC institutions needs further improvement. After taking into account the effect of external environmental factors and statistical noise, the SE of PHC institutions in HB, JX, AH, and SX showed a decrease, with SX experiencing the largest decrease, from 0.965 to 0.491. This result indicates that the SE of PHC institutions in SX is more influenced by environmental factors.

In the western region, PHC institutions in TB, QH, and NX have relatively low TE in the third stage, with all of these provinces obtaining values lower than 0.2. After excluding the effect of environmental factors and statistical noise, the TE of PHC institutions in TB, NX, and QH showed a significant decrease, which is due to the significant decline in SE. This result indicates that the SE of PHC institutions in TB, NX, and QH is more influenced by environmental factors. This phenomenon can be explained by the fact that TB, NX, and QH are less developed areas in China, and their external environment improved after the adjustment. Thus, they need to invest more health care resources to reach an efficient scale to improve their SE. In addition, the TE of PHC institutions in GZ declines the least, from 0.721 to 0.676, indicating a minimal influence of environmental factors.

In the northeast region, PHC institutions in LN, JL, and HLJ are relatively inefficient in the first and third stages, with PHC institutions in JL being the least efficient. The possible reasons for this phenomenon are the low level of economic development and the serious lack of quality medical and health professionals in the northeast region. The lack of dynamism in economic development and the low level of wages and social security in the northeast region have led to a serious brain drain problem, resulting in a lack of quality health professionals in PHC institutions [77]. The brain drain rates of graduates in LN, JL, and HLJ reached 15.56%, 40.65%, and 32.03%, respectively, which are higher than those in other provinces [76]. Among the three northeastern provinces, the brain drain of college graduates is more prominent in JL and is a critical reason for the lowest efficiency of PHC institutions in JL. In addition, affected by various factors such as the natural environment, the geographical environment, and economic and social development, the population loss in the in the northeast region is the most serious [78]. After taking into account the effect of external environmental factors and statistical noise, the PTE of PHC institutions in JL improves by 0.422, indicating that the PTE in JL under the influence of environmental factors is greatly undervalued.

**Fig. 7:**
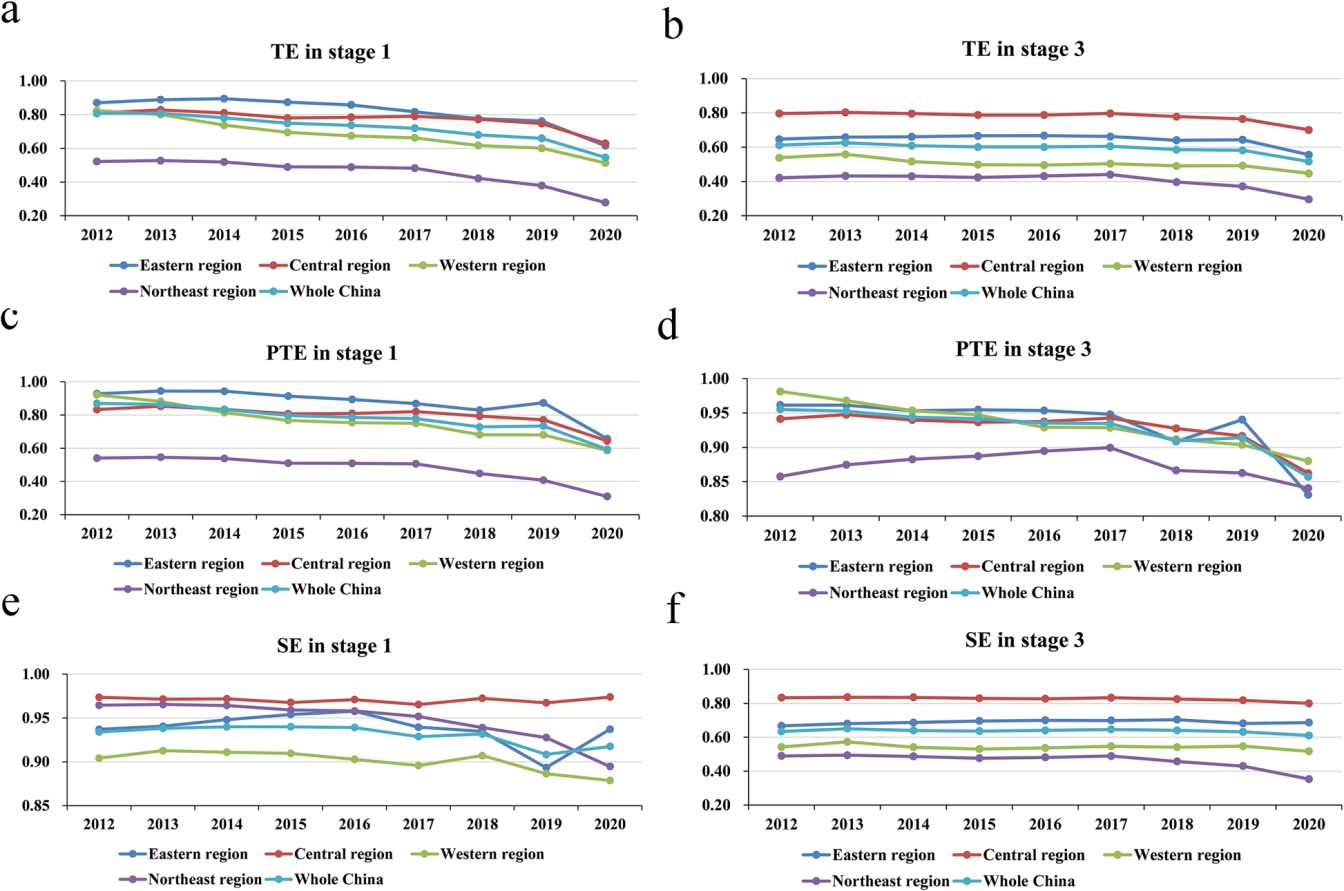
The trend in the efficiency of PHC institutions in the four regions from 2012 to 2020

As depicted in Fig. 7, the line graphs of the scores of the three types of efficiency in the four groups of regions calculated in the first and third stages reflect the tendency of efficiency. First, from the perspective of TE, the TE values of the four regions calculated in the first stage exhibit a pronounced downward trend over time, while the values calculated in the third stage display a relatively slight decrease, both indicating much room for efficiency improvement. The differences in the efficiency of PHC institutions between regions are evident, and the northeast region has remained in the most backward position, which is consistent with the conclusion reached by Yi et al. [53] when measuring the efficiency of health care facilities nationwide. The obvious difference in efficiency is likely due to the differences in the level of economic development and the resource endowment between the four regions. Second, from the perspective of PTE, the PTE values in the first and third stages exhibit a decreasing trend that fluctuates over time. This result demonstrates that there is still much room for improvement in PTE, and more effective measures should be taken to improve the medical technology and management of PHC institutions for greater efficiency. The PTE of PHC institutions in the northeast region remains the lowest, indicating that the levels of medical technology and management are the lowest. Third, compared to PTE, the SE values for the four regions are small in the third stage, and most of them are in a state of increasing returns to scale. This result indicates that the PHC institutions in the four regions have not yet reached an efficient scale and therefore have the potential to increase their investment scale. The SE of PHC institutions in the northeast region no longer lags significantly compared with that in other regions without considering environmental factors and random noise.

### Decomposition of the provincial TE of PHC institutions

**Fig. 8:**
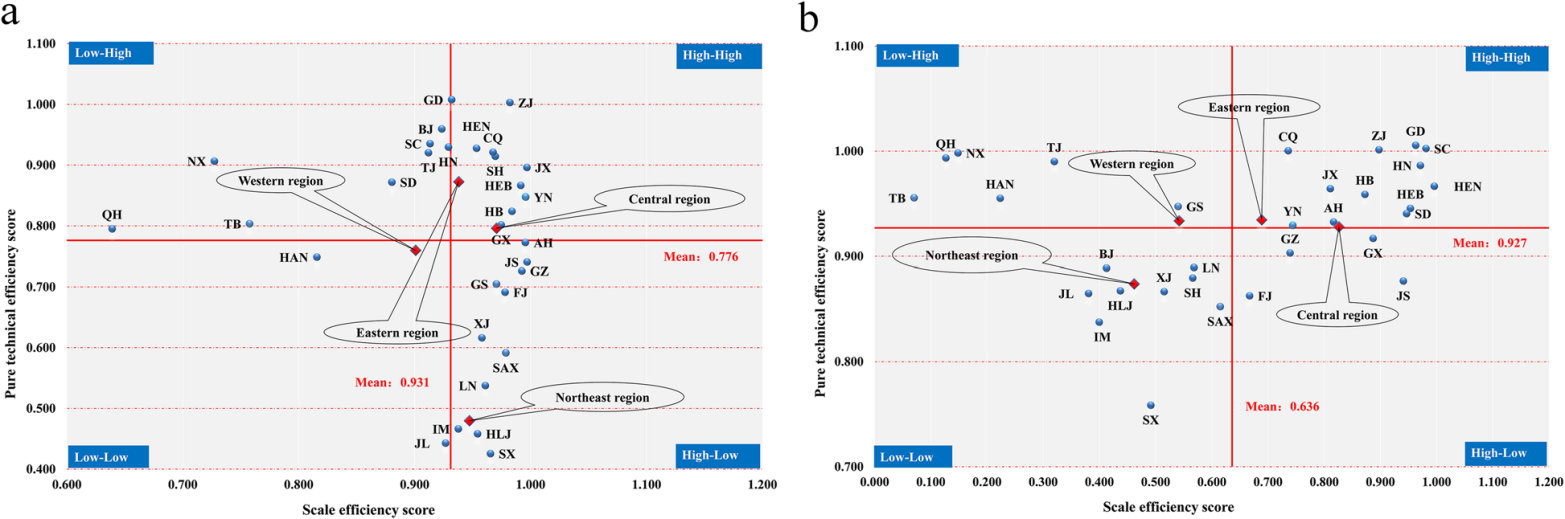
Categorization of the PTE and SE of 31 provinces. **a** and **b** denote the efficiency scores in stage 1 and stage 3, respectively

Figure 8 displays scatter plots based on the PTE and SE scores of PHC institutions in the first and third stages. Thirty-one provinces are classified into four categories based on the average scores of PTE and SE: high-high, low–high, low-low, and high-low categories. The two red lines in the scatter plots represent the average PTE and SE scores of PHC institutions.

The high-high category is composed of provinces with relatively high PTE and SE values of PHC institutions. The results of classification in the first stage show that nine provinces, i.e., ZJ, CQ, YN, SH, HEN, JX, HEB, HB, and GX, are in this category. When accounting for the effects of environmental factors and random noise, the PTE and SE values of PHC institutions in ZJ, CQ, YN, HEN, JX, HEB, and HB remain relatively high. The PTE values of PHC institutions in ZJ and GD are greater than 1 in the two stages. However, because they have SE values of less than 1, the PHC institutions in these two provinces do not reach the efficient state, implying that the PHC institutions in these two provinces can enhance their efficiency by increasing their SE. GD, SC, HN, and SD change from the low–high category to the high-high category, showing that the values of SE are significantly improved in a homogeneous environment.

The low–high category contains provinces with relatively high PTE values but low SE values. NX, QH, TB, and TJ are classified in this category, with or without considering the impact of environmental factors and random disturbances. This result implies that these four provinces can enhance the efficiency of their PHC institutions by improving SE. HAN, which originally belonged to the low-low category, is classified into the low–high category in the third stage, showing that the PTE of its PHC institutions is improved. However, the PHC institutions in this province still need to improve their SE. GS changes from the high-low category to the low–high category, indicating that the PTE of its PHC institutions is higher than that of some other provinces in a homogeneous environment. However, these institutions still need to improve their SE.

The low-low category consists of provinces whose PHC institutions have relatively low levels of both SE and PTE. JL falls into the low-low category in the first and third stages, indicating that among all provinces, the PTE and SE of PHC institutions in this province are relatively backward, which is probably related to the economic development level and inadequate policy support. HLJ, SX, LN, SAX, IM, and XJ change from the high-low category to the low-low category, indicating that the PTE and SE values of PHC institutions in these five provinces are relatively low. SH and BJ change from the high-high category and the low–high category, respectively, to the low-low category. As cities at the forefront of China's economic development, SH and BJ have a relatively privileged environment with a concentration of high-quality health care resources, advanced medical technology and quality management, resulting in their relatively high PTE values in the first stage. However, their PHC institutions do not outperform PHC institutions in other provinces in a homogeneous environment. All these provinces in the low-low category should focus on increasing both PTE and SE.

The high-low category includes provinces with relatively high SE values but relatively low PTE values. Whether or not the effects of environmental factors and random noise are considered, GZ, FJ, and JS remain in the high-low category, implying that low PTE is the main reason limiting the improvement in TE in these three provinces. GX changes from the high-high category to the high-low category. That is, in a homogeneous environment, PHC institutions in GX fall behind PHC institutions in other provinces in terms of PTE. Therefore, future efforts to improve the efficiency of PHC institutions in these provinces should be directed mainly toward improving medical technology and management.

## Conclusions

To achieve the goal of universal access to basic health care services, governments in China and elsewhere have placed great emphasis on improving the efficiency of PHC institutions. The Chinese government has also made substantial investments in PHC institutions and introduced initiatives to boost the efficiency of such institutions. However, little is known about the efficiency of PHC institutions in China. In addition, most studies have applied the traditional DEA model and two-stage DEA model to evaluate the efficiency of PHC institutions. These methods do not simultaneously take into account environmental factors, statistical noise, slack, the super-efficiency problem, and the comparability of efficiency values across periods, which can lead to biased results. Given these issues in DEA models, this study applies a modified three-stage DEA model that combines the advantages of the super-efficiency SBM DEA model, three-stage DEA model and GBT to measure the efficiency of PHC institutions in China, and it conducts a comprehensive analysis based on the empirical results. The following conclusions can be derived from this research:

1. Without considering the influences of external environmental factors and random noise, from 2012 to 2020, all three types of efficiency showed a downward trend. The average scores of TE, PTE, and SE were 0.721, 0.776, and 0.931, respectively. SE was obviously greater than PTE, indicating that PTE has great potential for improvement. Thus, for the government to enhance the efficiency of PHC institutions, greater efforts should be devoted to improving medical technology and management. Improving medical technology and management will help PHC institutions achieve better performance with equal investment.
2. The SFA regression models are significant in the second stage, demonstrating the need to eliminate the effects of external environmental factors and random noise to obtain more accurate results when comparing the efficiency of PHC institutions in different provinces. Of the external environmental variables selected for this study, both population density and residents’ annual average income have a significant negative impact on the input slack. It can be concluded that an increase in population density or in the average annual income of residents favors the efficiency of PHC institutions. The proportion of the population aged 65 and older has a positive effect on input redundancy and can therefore negatively influence the efficiency of PHC institutions. The implication is that an increase in the proportion of the population aged 65 and older is not conducive to the efficiency of PHC institutions. The number of people with a college education and above per 100,000 residents and the proportion of the urban population have a certain negative impact on the efficiency of PHC institutions. The proportion of the population aged 0–14 has both positive and negative effects on the slack in the input variables. Thus, its impact on the efficiency of PHC institutions needs more studies for verification. Per capita GDP has a certain positive impact on the efficiency of PHC institutions. It can be assumed that increasing GDP per capita will facilitate the efficiency of PHC institutions.
3. After removing the impact of external environmental factors and random disturbances, from 2012 to 2020, all three types of efficiency showed a downward trend, indicating that the efficiency of PHC institutions has not been improved in recent years. Rather, it has decreased, demonstrating that the measures and policies designed to strengthen the efficiency of PHC institutions need to be reviewed and readjusted. Additionally, PTE is significantly larger than SE, indicating that the most effective approach to improve the efficiency of PHC institutions is to expand the scale of investment. Considering that environmental factors exert a significant impact on the SE of PHC institutions, the influence of external environmental factors should be given due consideration when scaling up investments.
4. From the spatial perspective, there is a certain clustering phenomenon in the efficiency of PHC institutions. With the continuous promotion of regional economic development, medical association construction and regional medical center construction, the development of medical and health care institutions in an area is highly relevant to that in surrounding areas. In future studies, spatial factors can be given due consideration, and spatial measurement methodologies can be adopted for further investigation. Additionally, with or without taking into account external environmental factors and random noise, there are significant differences in the efficiency of PHC institutions among the four regions, with the lowest efficiency in the northeast region. Therefore, when formulating policies based on local conditions, appropriate consideration should be given to other neighboring regions to facilitate the coordinated development of interregional health care.

### Supplementary Information

**Additional file 1.**

## Data Availability

Please contact Peixi Wang to request data.

## Abbreviations

PHC: Primary health care
DEA: Data envelopment analysis
SBM: Slack-based measurement
GDP: Gross domestic product
COVID-19: Coronavirus disease 2019
DMUs: Decision-making units
IM: Inner Mongolia
GX: Guangxi
JL: Jilin
HLJ: Heilongjiang
GZ: Guizhou
YN: Yunnan
TB: Tibet
QH: Qinghai
NX: Ningxia
XJ: Xinjiang
SC: Sichuan
GS: Gansu
BJ: Beijing
TJ: Tianjin
HEB: Hebei
SH: Shanghai
JS: Jiangsu
ZJ: Zhejiang
FJ: Fujian
SD: Shandong
GD: Guangdong
HAN: Hainan
SX: Shanxi
AH: Anhui
JX: Jiangxi
HEN: Henan
HB: Hubei
HN: Hunan
CQ: Chongqing
SAX: Shaanxi
LN: Liaoning
SFA: Stochastic frontier analysis
GBT: Global benchmarking technique
MPI: Malmquist productivity index
CCR: Charnes, Cooper and Rhodes
BCC: Banker, Charnes and Cooper
TE: Technical efficiency
PTE: Pure technical efficiency
SE: Scale efficiency
LR: Likelihood ratio
