# Supplementary material for: Evaluating the efficiency of primary health care institutions in China: an improved three-stage data envelopment analysis approach
Source: BMC Health Serv Res. 2023 Sep 15;23:995. doi: 10.1186/s12913-023-09979-3 (PMC10503195; doi:10.1186/s12913-023-09979-3)
Supplement: Supplementary file 1 — Additional file 1. [file 12913_2023_9979_MOESM1_ESM.docx]

**Contents**

**Table S1.** Descriptive statistics of the data on the input‒output variables.

**Table S2.** Descriptive statistics of the data on the environmental variables.

**Table S1.** Descriptive statistics of the data on the input‒output variables

| Year | Variables | Beds | Doctors | Nurses | Other health workers | Treatment visits (ten thousand persons) | Admissions (ten thousand persons) |
| --- | --- | --- | --- | --- | --- | --- | --- |
| 2012 | Average | 42718 | 32567 | 17038 | 61272 | 13256 | 137 |
|  | Standard deviation | 32474 | 21834 | 11142 | 47190 | 10781 | 126 |
|  | Maximum | 125877 | 76709 | 45088 | 206054 | 41339 | 520 |
|  | Minimum | 2583 | 1185 | 356 | 6151 | 677 | 3 |
| 2013 | Average | 43545 | 33873 | 18601 | 60887 | 13949 | 139 |
|  | Standard deviation | 33017 | 23500 | 13131 | 45887 | 11336 | 126 |
|  | Maximum | 125964 | 89225 | 60213 | 198986 | 43861 | 497 |
|  | Minimum | 3087 | 1567 | 411 | 6207 | 722 | 3 |
| 2014 | Average | 44555 | 34327 | 19481 | 60281 | 14077 | 132 |
|  | Standard deviation | 33597 | 23735 | 13311 | 44978 | 11494 | 120 |
|  | Maximum | 128645 | 85987 | 56316 | 195028 | 43098 | 470 |
|  | Minimum | 3052 | 1826 | 518 | 6541 | 808 | 2 |
| 2015 | Average | 45608 | 35546 | 20858 | 59826 | 14006 | 130 |
|  | Standard deviation | 34665 | 24537 | 14174 | 43917 | 11407 | 119 |
|  | Maximum | 130741 | 86019 | 55688 | 189731 | 40759 | 454 |
|  | Minimum | 3198 | 1892 | 596 | 6371 | 798 | 2 |
| 2016 | Average | 46514 | 36949 | 22445 | 59399 | 14086 | 134 |
|  | Standard deviation | 35304 | 25403 | 15349 | 42468 | 11437 | 124 |
|  | Maximum | 132023 | 89428 | 63056 | 179359 | 39678 | 471 |
|  | Minimum | 3218 | 2017 | 701 | 6575 | 777 | 3 |
| 2017 | Average | 49307 | 39149 | 24813 | 59465 | 14287 | 144 |
|  | Standard deviation | 37346 | 26996 | 17058 | 41413 | 11623 | 133 |
|  | Maximum | 139395 | 95217 | 71700 | 172101 | 41582 | 519 |
|  | Minimum | 3659 | 2426 | 906 | 6436 | 939 | 3 |
| 2018 | Average | 51083 | 42100 | 27496 | 58299 | 14214 | 141 |
|  | Standard deviation | 38877 | 29507 | 19470 | 39901 | 11775 | 132 |
|  | Maximum | 143846 | 104967 | 82299 | 163906 | 42082 | 489 |
|  | Minimum | 3771 | 2586 | 1315 | 6980 | 946 | 3 |
| 2019 | Average | 52617 | 46343 | 30980 | 56890 | 14616 | 139 |
|  | Standard deviation | 40071 | 32511 | 21990 | 38258 | 12179 | 137 |
|  | Maximum | 148297 | 119863 | 90657 | 156104 | 43730 | 519 |
|  | Minimum | 3782 | 3372 | 1408 | 7037 | 960 | 2 |
| 2020 | Average | 53206 | 49561 | 34110 | 56321 | 13278 | 120 |
|  | Standard deviation | 40997 | 34529 | 23604 | 37347 | 10973 | 125 |
|  | Maximum | 149620 | 126763 | 97169 | 152910 | 36748 | 458 |
|  | Minimum | 3867 | 3233 | 1556 | 7197 | 930 | 1 |

**Table S2.** Descriptive statistics of the data on the environmental variables

| Year | Variables | Population density (per square kilometer) | Percentage of the population aged 0-14 (%) | Percentage of the population aged 65 and older (%) | Number of people with a college education and above per 100,000 residents | Proportion of the urban population (%) | Residents’ annual average income (yuan) | Per capita GDP (yuan) |
| --- | --- | --- | --- | --- | --- | --- | --- | --- |
| 2012 | Average | 141 | 16.46 | 9.40 | 9826 | 53.10 | 16756.74 | 42417.85 |
|  | Standard deviation | 680 | 3.99 | 1.66 | 6184 | 14.02 | 6832.45 | 19146.10 |
|  | Maximum | 3784 | 22.97 | 12.90 | 35574 | 89.30 | 37793.18 | 93569.52 |
|  | Minimum | 3 | 8.46 | 5.41 | 3782 | 22.87 | 8534.43 | 19102.87 |
| 2013 | Average | 142 | 16.41 | 9.68 | 10493 | 54.49 | 18310.80 | 46395.37 |
|  | Standard deviation | 694 | 3.80 | 1.77 | 6725 | 13.73 | 7555.45 | 20389.49 |
|  | Maximum | 3861 | 23.26 | 13.25 | 39267 | 89.60 | 42173.60 | 102425.60 |
|  | Minimum | 3 | 9.36 | 5.17 | 2111 | 23.93 | 9740.40 | 22265.58 |
| 2014 | Average | 143 | 16.49 | 10.06 | 10668 | 55.75 | 20167.10 | 49718.08 |
|  | Standard deviation | 701 | 3.85 | 1.94 | 6233 | 13.23 | 8192.38 | 21574.37 |
|  | Maximum | 3891 | 24.57 | 14.12 | 35975 | 89.30 | 45965.80 | 110055.49 |
|  | Minimum | 3 | 10.11 | 5.49 | 2283 | 26.23 | 10730.20 | 25200.95 |
| 2015 | Average | 144 | 16.52 | 10.47 | 12329 | 57.33 | 21966.20 | 52251.05 |
|  | Standard deviation | 699 | 4.07 | 1.89 | 6362 | 12.64 | 8842.37 | 22854.15 |
|  | Maximum | 3877 | 23.57 | 13.29 | 39543 | 88.53 | 49867.20 | 114928.35 |
|  | Minimum | 3 | 9.34 | 5.71 | 6383 | 28.87 | 12254.30 | 26913.67 |
| 2016 | Average | 145 | 16.64 | 10.85 | 11959 | 58.84 | 23821.00 | 56026.63 |
|  | Standard deviation | 702 | 3.93 | 2.12 | 7267 | 12.13 | 9585.12 | 25129.71 |
|  | Maximum | 3891 | 24.06 | 13.97 | 42069 | 89.00 | 54305.30 | 123945.88 |
|  | Minimum | 3 | 9.67 | 4.98 | 4638 | 31.57 | 13639.20 | 28572.90 |
| 2017 | Average | 146 | 16.80 | 11.39 | 12793 | 60.24 | 25973.80 | 60505.25 |
|  | Standard deviation | 701 | 3.97 | 2.27 | 7697 | 11.67 | 10397.01 | 26969.45 |
|  | Maximum | 3889 | 23.99 | 14.28 | 44069 | 89.10 | 58988.00 | 131554.54 |
|  | Minimum | 3 | 9.95 | 5.79 | 6940 | 33.38 | 15457.30 | 29579.30 |
| 2018 | Average | 146 | 16.86 | 11.94 | 12938 | 61.50 | 28228.00 | 65084.74 |
|  | Standard deviation | 703 | 3.95 | 2.46 | 7601 | 11.39 | 11278.96 | 28330.16 |
|  | Maximum | 3903 | 23.54 | 15.16 | 45181 | 89.13 | 64182.60 | 138321.08 |
|  | Minimum | 3 | 9.84 | 5.68 | 6400 | 33.80 | 17286.10 | 32787.55 |
| 2019 | Average | 147 | 16.78 | 12.57 | 13472 | 62.71 | 30732.80 | 69877.82 |
|  | Standard deviation | 704 | 4.27 | 2.59 | 7617 | 11.16 | 12165.94 | 30576.24 |
|  | Maximum | 3913 | 26.07 | 16.26 | 46529 | 89.22 | 69441.60 | 161512.69 |
|  | Minimum | 3 | 9.97 | 6.02 | 7117 | 34.51 | 19139.00 | 34748.11 |
| 2020 | Average | 147 | 17.97 | 13.52 | 15383 | 63.89 | 32188.80 | 71694.69 |
|  | Standard deviation | 706 | 4.14 | 2.87 | 6535 | 10.88 | 12455.13 | 30762.48 |
|  | Maximum | 3924 | 24.53 | 17.42 | 41986 | 89.30 | 72232.40 | 164927.14 |
|  | Minimum | 3 | 9.80 | 5.67 | 10792 | 35.73 | 20335.10 | 36052.38 |
